# The assessment of *Pseudomonas aeruginosa* lectin LecA binding characteristics of divalent galactosides using multiple techniques

**DOI:** 10.1093/glycob/cwab074

**Published:** 2021-07-10

**Authors:** Pouya Zaree, Javier Sastre Torano, Cornelis A M de Haan, Richard A Scheltema, Arjan Barendregt, Vito Thijssen, Guangyun Yu, Frits Flesch, Roland J Pieters

**Affiliations:** Department of Chemical Biology and Drug Discovery, Utrecht Institute for Pharmaceutical Sciences, Utrecht University, Universiteitsweg 99, 3584 CG Utrecht, the Netherlands; Department of Chemical Biology and Drug Discovery, Utrecht Institute for Pharmaceutical Sciences, Utrecht University, Universiteitsweg 99, 3584 CG Utrecht, the Netherlands; Section Virology, Division of Infectious Diseases & Immunology, Department of Biomolecular Health Sciences, Faculty of Veterinary Medicine, Utrecht University, Yalelaan 1, 3584 CL Utrecht, the Netherlands; Biomolecular Mass Spectrometry and Proteomics, Bijvoet Center for Biomolecular Research and Utrecht Institute of Pharmaceutical Sciences, Utrecht University, Padualaan 8, 3584 CH Utrecht, the Netherlands; Netherlands Proteomics Centre, Padualaan 8, 3584 CH Utrecht, the Netherlands; Biomolecular Mass Spectrometry and Proteomics, Bijvoet Center for Biomolecular Research and Utrecht Institute of Pharmaceutical Sciences, Utrecht University, Padualaan 8, 3584 CH Utrecht, the Netherlands; Netherlands Proteomics Centre, Padualaan 8, 3584 CH Utrecht, the Netherlands; Department of Chemical Biology and Drug Discovery, Utrecht Institute for Pharmaceutical Sciences, Utrecht University, Universiteitsweg 99, 3584 CG Utrecht, the Netherlands; Department of Chemical Biology and Drug Discovery, Utrecht Institute for Pharmaceutical Sciences, Utrecht University, Universiteitsweg 99, 3584 CG Utrecht, the Netherlands; Department of Chemical Biology and Drug Discovery, Utrecht Institute for Pharmaceutical Sciences, Utrecht University, Universiteitsweg 99, 3584 CG Utrecht, the Netherlands; Department of Chemical Biology and Drug Discovery, Utrecht Institute for Pharmaceutical Sciences, Utrecht University, Universiteitsweg 99, 3584 CG Utrecht, the Netherlands

**Keywords:** binding kinetics, LecA inhibition, multivalency, protein–carbohydrate interactions, residence time

## Abstract

*Pseudomonas aeruginosa* is a widespread opportunistic pathogen that is capable of colonizing various human tissues and is resistant to many antibiotics. LecA is a galactose binding tetrameric lectin involved in adhesion, infection and biofilm formation. This study reports on the binding characteristics of mono- and divalent (chelating) ligands to LecA using different techniques. These techniques include affinity capillary electrophoresis, bio-layer interferometry, native mass spectrometry and a thermal shift assay. Aspects of focus include: affinity, selectivity, binding kinetics and residence time. The affinity of a divalent ligand was determined to be in the low-nanomolar range for all of the used techniques and with a ligand residence time of approximately 7 h, while no strong binding was seen to related lectin tetramers. Each of the used techniques provides a unique and complementary insight into the chelation based binding mode of the divalent ligand to the LecA tetramer.

## Introduction

Lectins are carbohydrate-binding proteins with diverse functions that are found in all domains of life (Chemani et al. 2009); (Huang et al. 2018). These proteins play crucial roles in various processes such as cell–cell recognition, infection processes and immune defense (Lee and Lee 1995); (Brown et al. 2018); (Bernardi et al. 2013). They are generally characterized by an intermediate to low affinity toward their carbohydrate ligands, a limitation that is frequently overcome in nature through multivalency of both the lectin receptors and their carbohydrate ligands, resulting in enhanced binding or inhibition (Cecioni et al. 2015); (Fasting et al. 2012); (Wittmann and Pieters 2013). Lectins are involved in the infection process of the Gram-negative bacterium *Pseudomonas aeruginosa*, an important member of the often highly drug-resistant ESKAPE pathogens, which include *Enterococcus faecium*, *Staphylococcus aureus, Klebsiella pneumoniae, Acinetobacter baumannii, P. aeruginosa and Enterobacter* species, and currently cause most of the extreme hospital infections in western countries (Boucher et al. 2009); (Cioci et al. 2003). The two bacterial lectins LecA and LecB, are virulence factors that are important for bacterial adhesion and biofilm formation of *P. aeruginosa* (Avichezer et al. 1992), (Chemani et al. 2009). LecA is reported to bind to galactosyl residues presented on the lung epithelial cell membrane, leading to lung injury. Subsequently, inhibition of LecA has been examined by numerous studies as a novel approach to control *P. aeruginosa* infection (Chemani et al. 2009); (Goyard et al. 2019).

**Fig. 1:**
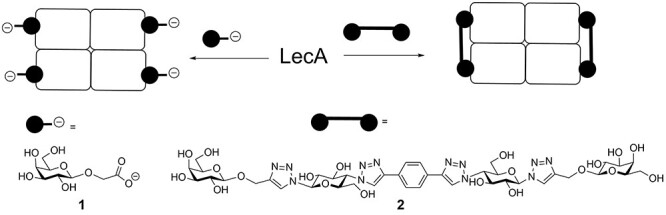
Structures and binding modes of mono- and divalent LecA inhibitors **1** (500 μM) and **2** used in this study.

**Fig. 2:**
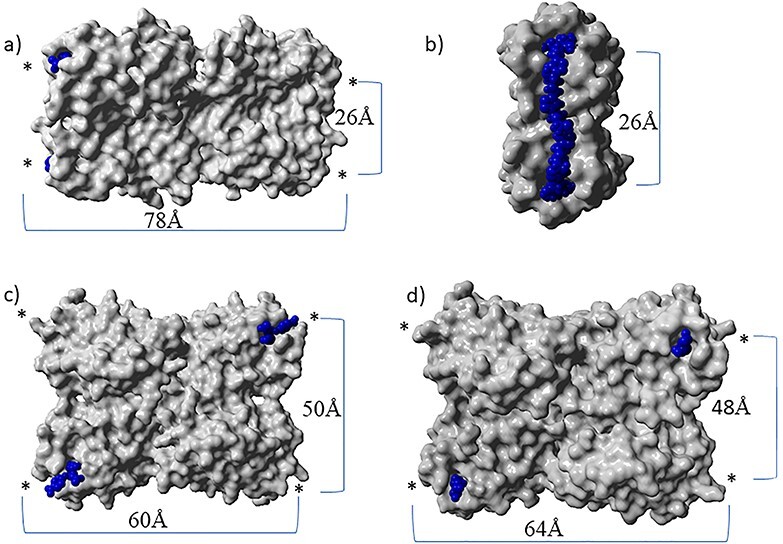
(A) Side view of the LecA tetramer and indication of the distances between galactoside binding sites, measured from the anomeric oxygens. An asterisk indicates the position of a binding site, even if it is not visible from this side. (PDB 1OKO). (B) X-ray structure of a divalent ligand bridging two binding sites (PDB 4YWA) (Visini et al. 2015). (C) Jacalin with bound galactosides (PDB 1UGW). (D) *Maclura pomifera* Lectin (MPL) with bound galactosides (PDB 3LLZ).

Recently we prepared divalent ligands as inhibitors for LecA. Bridging two binding sites that were separated by 26 Å, enabled simultaneous binding of two galactose moieties, which dramatically increased the binding affinity (Yu et al. 2019b); (Visini et al. 2015); (Pertici et al. 2013). An alternating motif of glucose, triazole and aryl groups was shown to have the right mix of rigidity, solubility and ease of synthesis to function as effective spacers between the two terminal galactose units (Figure 1). Spacers were changed with respect to the center unit as well as the aglycon portions in an attempt to optimize dynamics and improve interactions with the protein. Affinities of the divalent ligands were measured by isothermal titration calorimetry (ITC), and K_D_’s as low as 12 nM were determined, notably for a compounds with either a rigid (phenyl) or flexible (n-butyl) unit at the core (Yu et al. 2019b). Although it was clear that by optimization of the molecular structure close to optimal divalent ligands were obtained (Pertici et al. 2013); (Visini et al. 2015), it was not experimentally established whether these ligands can be considered selective. To be complete, we define selectivity in two ways. Firstly, are the divalent ligands preferred over other mono- and multivalent galactoside ligands? The answer to this question is largely known, because of the development of the compounds in the past and the many K_D_ measurements. Secondly, does strong binding of the optimized divalent LecA-ligand only occur to LecA, even when compared to lectins with similar monovalent galactoside binding strength and similar multivalency but with other distances between the binding sites. To this end we used two additional galactophilic tetrameric lectins. The first was *Maclura pomifera* Lectin (MPL), a tetrameric plant protein (44 kDa) that consists of four subunits with a high affinity for the tumor-associated T-antigen disaccharide and many O-linked glycopeptide structures (Sarkar et al. 1981); (Shimura and ichi 1997) (Figure 2). Its binding sites are separated by 48 Å and 64 Å The second is Jacalin, a plant-based galactose binding lectin found in Jackfruits with a tetrameric two chain structure (Tachibana et al. 2006); (Smith et al. 2003) and distances between the binding sites of 50 Å and 60 Å.

In order to determine the selectivity, a number of analytical techniques were used as detailed below. Besides the selectivity issues, also other noteworthy properties of drug compounds are addressed, most notably the residence time (Hoare et al. 2020), an important parameter for drug molecules, which we anticipated to dramatically increase for divalent ligands. Residence time depends on binding kinetics and can be measured by any technique that allows this, such as SPR. The residence time (1/k_off_) is a measure for the duration of target occupancy and a long residence time is linked to successful drug development. Furthermore slow binding kinetics may lead to overlooking good compounds in the discovery process that have not yet reached equilibrium. Affinity capillary electrophoresis (ACE) is an effective analytical tool to study ligand–protein interactions (Chang et al. 2005). This technique is based on the notion that the effective electrophoretic mobility (μ_eff_) of the protein–ligand complexes varies from the μ_eff_ of the intact proteins or ligands (Aizpurua-Olaizola et al. 2018); (Olabi et al. 2018). Capillary electrophoresis (CE) has the intrinsic capacity for the separation of a small amount of analyte in complex samples and it has numerous merits such as high separation efficiency, high sensitivity, high speed and low cost (Geng et al. 2018). Bio-layer interferometry (BLI) is a label-free method based on the real-time optical monitoring of biomolecular interactions (Yang et al. 2015). Experiments are performed in standard multiwell plates containing a solution of one partner in which a biosensor tip is immersed that is covalently functionalized with the second partner. Tips are composed of a biocompatible layer to minimize nonspecific interactions with the sensor and a thin layer coated with reactive groups (Shah and Duncan 2014). Upon irradiation of the functionalized biosensor with a white light laser, the detection of interferometry variation occurring during association/dissociation steps allows for the determination of kinetic and thermodynamic parameters of the interaction, with comparable precision to other physicochemical techniques (Laigre et al. 2018). The fluorescence-based thermal shift assay (TSA) is a common technique for the identification of ligands and inhibitors of target proteins from compound libraries (Zubrienė et al. 2009). It utilizes a fluorescent dye, sensitive to its environment, to screen protein thermal unfolding. The relative ligand-binding affinity can be deduced from the shift of the unfolding temperature (ΔT_m_) in the presence of ligands relative to that obtained in the absence of ligands (Lo et al. 2004). Native mass spectrometry (MS), allows for proteins in their native or near-native states in solution to be introduced into the gas phase and interrogated by mass spectrometry (Leney and Heck 2017). This provides insights into the stoichiometry of complexes (Zhang et al. 2013); (Göth and Pagel 2017) providing highly complementary information to the other techniques. Using these techniques we show that the divalent ligand interacts with LecA specifically with high affinity and a long residence time. Considering limited data are available for the binding mode and binding kinetics of potent glycoligands as described here, the data may help to pave the way for their use as probes and therapeutics.

## Results

### Affinity capillary electrophoresis

For affinity studies, two different LecA ligands were used. The first was the carboxylated galactoside (**1**). This was used as the reference monovalent ligand and its negatively charged carboxylate increases the chances of a protein shift in CE where charges are the important mobility determinant. Furthermore we used the bivalent carbohydrate (**2**), previously optimized for bivalent binding (Yu et al. 2019b). These glycoligands were added to the optimized CE background electrolyte (BGE) and the protein LecA was injected. For *K*_D_ determination the BGE containing increasing concentrations of carbohydrate **1** or carbohydrate **2** were prepared and used for the analysis of LecA (10 μM). The carbohydrate concentrations within the BGE were varied between the low-nanomolar range to the micromolar range to cover a large spectrum of possible *K*_D_’s. For each carbohydrate and each concentration, the difference of the measured μ_eff_ of LecA with the μ_eff_ acquired without carbohydrate in the BGE, was determined. The obtained values were plotted vs. the carbohydrate concentration, and fitted using nonlinear regression. LecA analysis was optimized for high μ_eff_ values to enhance mobility shifts upon ligand binding, thus enabling *K*_D_ determination. To this end, different BGEs containing ammonium acetate, potassium dihydrogen phosphate or ammonium formate at concentrations ranging from 10 to 100 mM with a pH range of 2–12 were studied. Potassium dihydrogen phosphate was selected because its use resulted in reproducible μ_eff_ values at 25 mM and pH 7. One of the initial problems was poor protein separation due to adsorption of the proteins onto the capillary wall. To prevent this, capillaries were coated with Ultra Trol^LN^ dynamic precoating. As the p*I* of LecA is 4.8, the overall charge of LecA at pH 7 is negative so reverse polarity was selected.

**Fig. 3:**
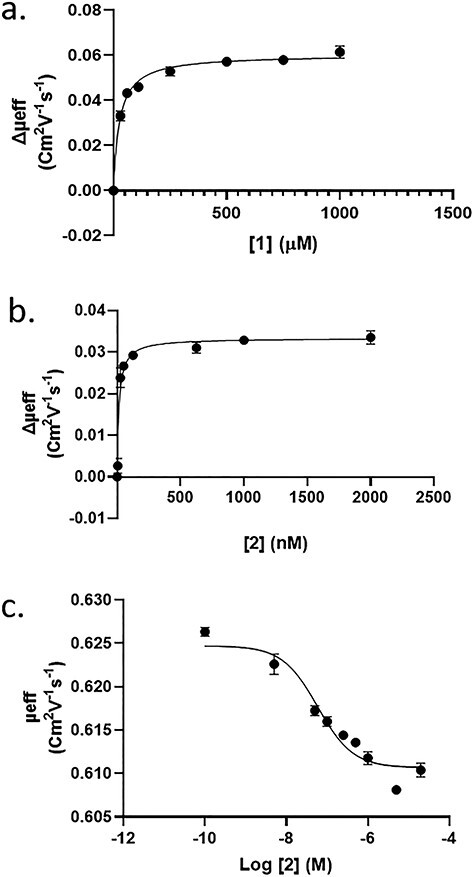
ACE binding curves for LecA-carbohydrate complexes obtained by plotting μ_eff_ shifts against the concentration of carbohydrates in the BGE. Carbohydrate **1** (A), carbohydrate **2** (B) and in a competition experiment where carbohydrate **1** is displaced by **2** (C).

Using our optimized conditions, a *K*_D_ value of 27 μM was obtained for carbohydrate **1** and a *K*_D_ value of 16 nM for carbohydrate **2** in direct binding experiments. The *K*_D_ value for **2** was reproducible despite the fact that it is a neutral ligand. In addition we performed a competition experiment where the neutral divalent **2** displaces the charged monovalent **1**. In this case, a *K*_D_ value of 6 nM was determined (Figure 3).

**Fig. 4:**
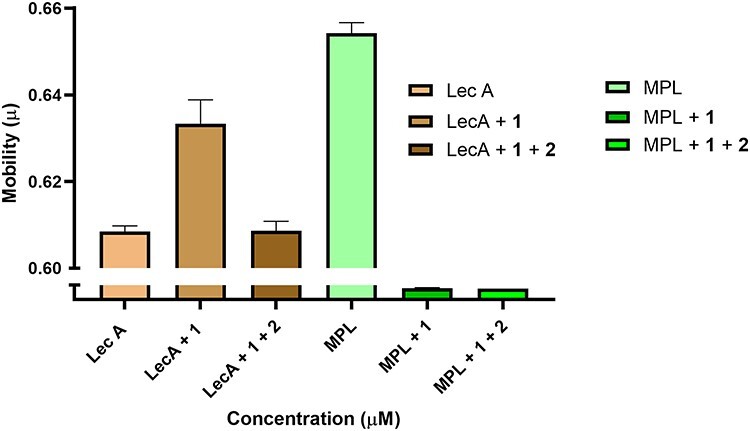
CE analysis of LecA (10 μM) and MPL (62.5 μM) using UltraTrol LN-coated capillary and a BGE of 25 mM KH_2_PO_4_ (pH 7) containing 1 mM of monovalent galactoside, 20 μM of divalent galactosides, or no sugar at all.

The selectivity of carbohydrate **2** toward LecA was demonstrated using the MPL as a binding competitor (Figure 4). A mixture of LecA and MPL was analyzed by ACE, and affinity studies were carried out by adding the carbohydrates **1** and **2** to the BGE. As shown in Figure 3, LecA and MPL yielded distinct μ_eff_ values. When carbohydrate **1** was added to the BGE, both galactophilic tetrameric lectins showed a sizeable shift in μ_eff_ values in agreement with binding of a charged ligand. When a small amount of neutral divalent **2** was added as a higher affinity competitor, the LecA peak shifted back to a μ_eff_ value close to that observed in the absence of any carbohydrates and consistent with binding of a neutral ligand. MPL, however, showed a different behavior as the divalent **2** was unable to displace monovalent **1**, based on the observed μ_eff_ values, consistent with divalent **2** binding MPL with a similar affinity as monovalent **1**. Furthermore, whereas LecA in combination with divalent **2** gave a small but significant shift in mobility, MPL did not show this under the same conditions.

**Fig. 5:**
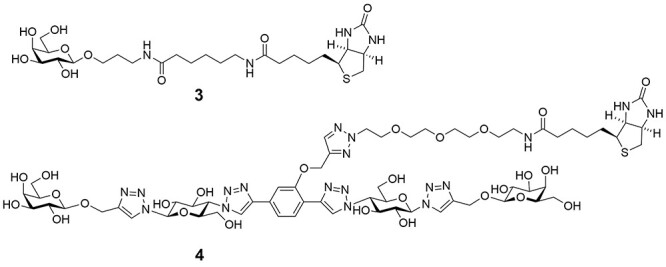
Structures of biotinylated mono- and divalent LecA inhibitors used in BLI.

### Bio-layer interferometry

**Fig. 6:**
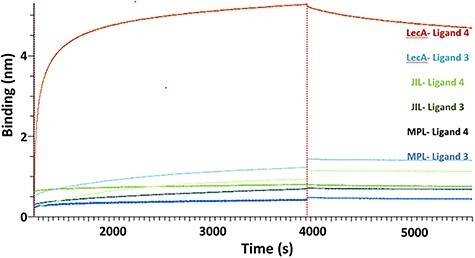
Biolayer interferometric analysis of binding and dissociation of LecA, MPL and JIL (all at 10 μM) to a streptavidin sensor displaying either monovalent **3** or divalent **4**. Curves shown display loading of **3** and **4** (<6000 s), association (6000–7500 s) and dissociation (>7500 s) parts which are shown as MPL- Ligand **3**, MPL-Ligand **4**, JIL-Ligand **3**, JIL-Ligand **4**, LecA-Ligand **3** and LecA-Ligand **4**.

The biotinylated galactoside **3** (Figure 5) was immobilized on a streptavidin coated sensor. The galactophilic tetrameric lectins LecA, MPL and JIL (Figure 2), were added to the sensor at 1 μM, however no detectable signal was observed with these lectins. This is likely due to the well-known low affinity of monovalent ligands for most lectins even though the presentation of the ligand on the chip was also multivalent, but apparently not matching the distances between binding sites. Next, the binding properties of the divalent biotinylated **4** with the mentioned lectins were evaluated. The sensorgrams with this compound are shown in Figure 6. The interaction between LecA and compound **4** could be observed and was shown to be reversible. Indeed, during the dissociation step, the signal only partially decreases down to the baseline and this lack of full dissociation indicates high affinity. Using this setup, kinetic parameters of the interaction were determined for the divalent galactoside to evaluate whether the divalent binding mode of this ligand influences the lectin–ligand kinetics. By fitting the sensorgrams with a global model (2: 1), association (k_a_ or k_on_) and dissociation (k_d_ or k_off_) constants were determined and the K_D_ was calculated from the ratio of k_d_ over the k_a_ while the residence time (τ) was derived from 1/k_d_. As mentioned, no significant association and dissociation phenomena were observed between compound **3** and the lectins LecA, MPL and JIL at concentrations of the lectins of up to 10 μM. The same was true for compound **4** and lectins MPL and JIL. However, a K_D_ of 31 nM was determined for compound **4** bonding to LecA, along with an approximation of the residence time of 7 h with LecA (Table I).

**Table I:** Kinetic parameters of interaction between LecA and compound **4** based on Figure 6

	LecA + compound **4**
k_on_(M^−1^ s^−1^)	1.22 × 10^3^ ± 94.1
k_off_(s^−1^)	3.83 × 10^−5^ ± 9.86× 10^−7^
K_D_ (M)	3.14 × 10^−8^ ± 8.44 × 10^−10^
τ (s)	26110 (7.3 h)

### Thermal shift assay

**Fig. 7:**
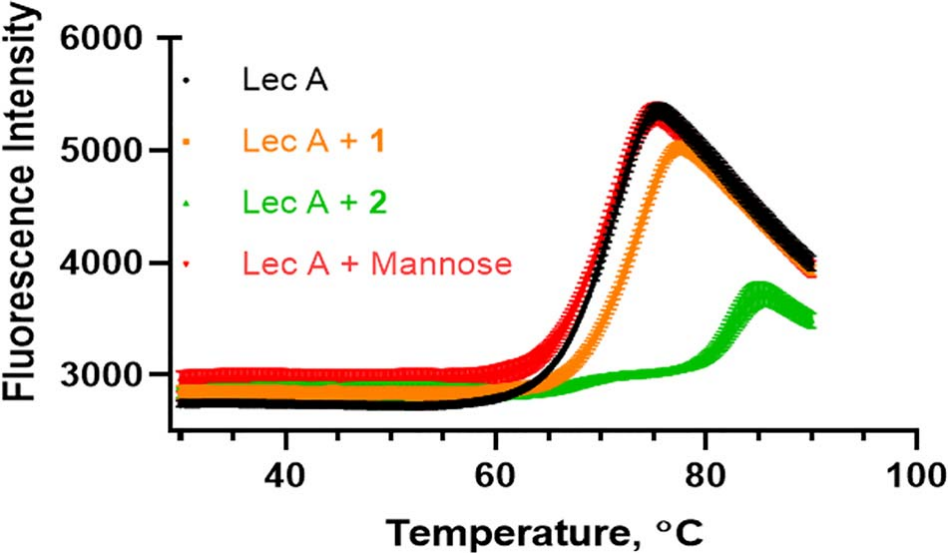
Denaturation curves of fluorescence-based thermal shift assays of LecA (20 μM) in PBS (pH 7.4) with compound **2** (20 μM), D-Mannose and compound **1** (500 μM).

As ligand binding is known to shift the melting point of proteins in a ligand concentration dependent manner, we performed the TSA experiments with two different concentrations of LecA (0.5 and 1 mg/mL) containing the fluorescent dye sypro orange. The higher concentrations of LecA showed a higher fluorescence signal than the lower, but the melting temperatures were the same for both concentrations. Therefore, we selected a fixed concentration of 1 mg/mL for the subsequent experiments. Compound **1** was found to stabilize the protein only by about 2.3°C at a ligand concentration of 500 μM. Divalent **2** stabilizes LecA by more than 11°C at a concentration of 20 μM (Figure 7). Nonbinding ligand D-mannose did not stabilize LecA at all at 500 μM as a negative control (Table II).

**Table II:** Melting temperature of free and ligand-bound unfolding of LecA

[Ligand], μM	T_m_, °C
No ligand	71.7
[D-Mannose] 500 μM	71.7
[Compound **1**] 500 μM	74.0
[Compound **2**] 20 μM	82.7

### Native ESI–MS

**Fig. 8:**
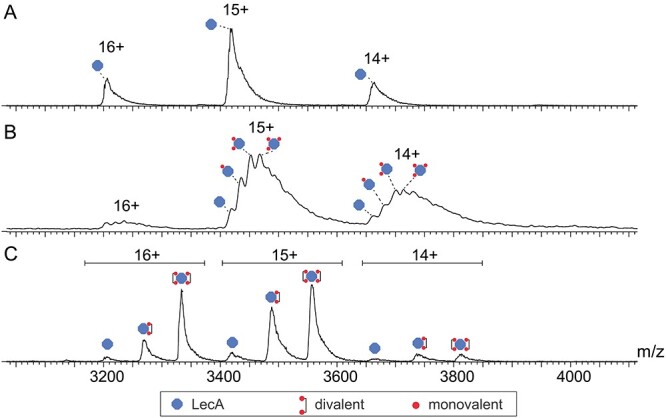
Positive nano ESI–MS spectra of 10 μM LecA (A) with 600 μM monovalent Gal **1** (B) and 2 μM divalent Gal **2** (C).

To uncover the occupancy of binding of monovalent **1** and divalent **2**, we employed MS, which revealed 3 isoforms for LecA, all detected at charge states 14+, 15+ and 16+. The neutral deconvolution of LecA revealed a mass of 51199.49 Da, matching the theoretical mass of 51200 Da well (Figure 8a). At the high concentration of monovalent compound **1** (600 μM) incubated with LecA (10 μM), a varying number of binding pockets of the tetrameric lectin were occupied by the monovalent ligand indicated by four different peaks in close proximity (Figure 8b). At these concentrations, an estimated number of copies of LecA of 8% remains fully unoccupied, 18% has one pocket occupied, 26% has two pockets occupied, 27% has three pockets occupied and 21% is fully occupied. We used the intensity values as determined by mass spectrometry as a proxy of their abundances in solution to calculate the occupancy values, for which previously it was shown that there is good correlation (Seo et al. 2016).

**Fig. 9:**
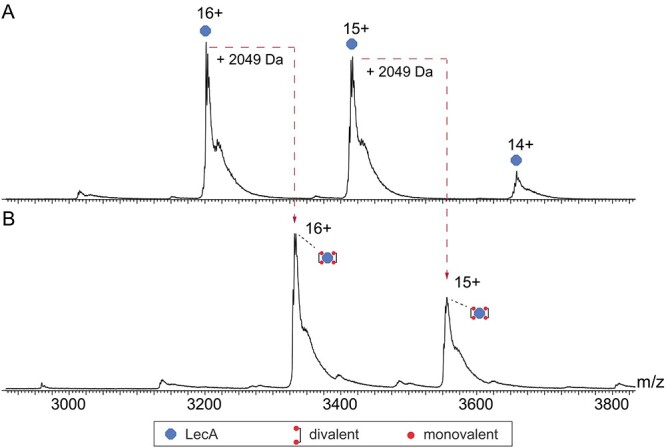
Positive nano ESI–MS spectra of 10 μM LecA without sugar (A) and with 10 μM divalent Gal **2** (B).

At a low (substoichiometric) concentration of divalent compound **2** (2 μM), 3 m/z peaks were observed that correspond to unbound LecA (estimated 6%), LecA bound to one compound **2** (estimated 39%) and LecA bound to two of the divalent ligand **2** (estimated 55%) (Figure 8c). Repeating the measurement with a higher concentration of the divalent ligand (10 μM, 2 equiv. considering the 2:1 binding mode), we detected only fully occupied LecA tetramer, i.e. bound to two divalent ligands (Figure 9), interestingly, with notable suppression of the 14+ ionization state. As no peaks were detected corresponding to a single or triple binding pockets of the tetrametric lectin, excellent support is provided for the effective divalent binding of our molecule at reasonable concentrations.

## Discussion

In this study, we assessed the different characteristics of divalent galactoside inhibitors for LecA using a range of analytical techniques, that each provides complementary insights into the binding event with respect to selectivity and binding kinetics. Using ACE, selectivity and specificity of a divalent ligand for lectin was first tested. As the divalent carbohydrates are neutral at physiological pH the introduction of a charged group was deemed necessary in order to be able to detect the binding event. The LecA peak shifted upon binding of a monovalent charged ligand. Addition of a stronger binding divalent ligand reversed the effect through competition, demonstrating effective binding. The latter observation could not be made for the related lectin MPL whose binding site separation do not match those of **2**, demonstrating the selectivity of the optimized divalent galactoside toward LecA.

In the BLI assay, experiments confirmed the low-nanomolar binding of compound **2** to LecA and showed for the first time the long residence time that can result from the divalent binding mode. Recently, a related divalent ligand was reported with a similar K_d_ of ca. 11 nM (Zahorska et al. 2020). In this study, both the on- and off-rates of the binding kinetics of this compound were determined by SPR and found to be ca. 2 orders of magnitude faster than those for **2**. Based on these data a residence time on the order of 10 min can be determined, i.e. considerably shorter than the 7 h we determined for **4**. The only significant difference is that we immobilized the divalent ligand instead of the protein in the SPR study. A possible cause of these different numbers in this very relevant comparison is the fact that our spacers contain significantly hydrated glucose units which help solubility but may slow mobility and increase the residence time, in comparison to the more aromatic and hydrophobic spacer of the mentioned 11 nM compound. Unfortunately no direct measurement of the residence time of the monovalent ligands was possible, because of the weak binding and fast kinetics (Zahorska et al. 2020); (Jayaraman 2009). Recently, residence times of monovalent glycomimetics for siglec showed a residence time of ca. 10 s (Kroezen et al. 2020), whereas glycomimetic lecB ligands were able to increase residence time from 45 s of the natural sugars to 5–20 min for glycomimetics ligands (Sommer et al. 2015). Our experiments clearly indicate that the affinity increase due to multivalency is accompanied by a similar increase in residence time. Whether it results in disproportionate residence time increases is too soon to conclude, even though conceptually this is likely due to the fact that the ligand is held at two positions on the protein. The experiments relied on streptavidin to immobilize the ligands. This protein contains four binding sites and two neighboring monovalent ligands bound to streptavidin, could have been a suitable divalent arrangement for LecA. We saw no evidence of such a suitable divalent ligand presentation as all signals involving **3** remained close to the baseline.

We additionally studied the effect of inhibitors on protein–carbohydrate complex stability via TSA. Compound **1** is a relatively weak monovalent ligand and had a small effect on the protein stability. High affinity ligand **2** considerably increased the stability of LecA, which can be rationalized by considering that the divalent ligand bridges two protein units of the tetramer.

In native ESI–MS, we investigated the binding mode of divalent inhibitor **2**. It clearly confirmed the mode in which two bivalent ligands bridge two subunits of the tetrameric LecA. Clear signals were observed, even at low stoichiometry, in agreement with strong binding, and with no evidence of higher aggregations, consistent with previous studies. (Yu et al. 2019a).

Concluding, the use of all the techniques has given us unique insights into the two to one binding mode of the divalent galactoside ligand to the tetrameric LecA. All techniques confirm the very strong binding in the low-nanomolar range vs. the micromolar range of the monovalent ligand. ACE does so in a direct binding as well as in a competition assay displacing a charged monovalent ligand. ACE also shows that the divalent ligand needs the correct spacing of binding sites to achieve strong binding. BLI confirms that as indeed no significant binding is observed for MPL and JIL, both tetrameric lectins that bind monovalent galactosides with similar affinities, but have different distances between those binding sites than LecA. BLI also enabled to identify the long residence time. Finally the TSA showed the large stabilizing effect of a divalent ligand and the native MS confirmed the binding mode with no indications of any alternatives involving bridging to other LecA tetramers or more extensive aggregation. The work has now validated several techniques for future studies of multivalent carbohydrate ligands and also our results show that relatively long-distance divalent ligands containing weakly binding sugars can act as selective and potent and thus well-behaved molecular probes and have potential as therapeutics.

## Materials and methods

### Protein and reagents

Lectin LecA from *Pseudomonas aeruginosa* (≥70%) and Jacalin were obtained from Sigma–Aldrich (Steinheim, Germany). MPL were obtained from Vector Laboratories (San Francisco, CA, USA). Precoating (N-substituted acrylamide copolymer) were obtained from Target Discovery (Palo Alto, CA, USA). The fluorescent dye Sypro orange was purchased from Sigma–Aldrich (St. Louis, USA) and α-D-Galactose-sp-biotin from CarbosyntH Ltd (UK). The other studied carbohydrates were synthesized and purified in our laboratories (Yu et al. 2019b).

### Capillary electrophoresis

CE analyses were carried out using an Agilent 7100 instrument (Agilent technologies, USA). Bare fused-silica capillaries, with a 100 μm internal diameter, 365 μm outer diameter and a 50 cm total length, were from Biotaq (Gaithersburg, MD, USA). The detection window of the capillary was created at 10 cm from the outlet of the capillary. Hydrodynamic injections were performed at 17 mbar for 4 s, by injecting LecA or MPL (10 μM). The separation voltage was set at −10 kV and the caplillary temperature was maintained at 25°C during analysis. Ultraviolet detection was performed from 190 nm to 280 nm, but data recorded at 214 nm were used in this study. The BGE for the analysis of the lectins was potassium phosphate prepared by dissolving the appropriate amount of compounds in water and adjusting the pH with a sodium hydroxide solution. Capillaries were coated with Ultra Trol LN. Stable currents of 45 μA were observed during the affinity measurements with optimum buffer conditions (25 mM KH_2_PO_4_ at pH 7).

### Affinity CE

In order to study the affinity of LecA toward inhibitors, LecA was injected and carbohydrate inhibitors were added to the BGEs. BGEs were prepared containing 1 mM of compound **1**, 20 μM of compound **2**, or without carbohydrate. A 10 μM solution of LecA was injected in all cases and μ_eff_ shifts were optimized. The μ_eff_ were experimentally obtained using equation 1:

(1) \begin{equation*} {\mu}_{\mathrm{eff}}=\frac{{\mathrm{L}}_{\mathrm{t}}{\mathrm{L}}_{\mathrm{d}}}{\mathrm{V}}\left(\frac{1}{{\mathrm{t}}_{\mathrm{P}}}\right)\left[{\mathrm{cm}}^2{\mathrm{V}}^{-1}{\mathrm{s}}^{-1}\right] \end{equation*}

where L_t_ is the total capillary length, L_d_ is the capillary length to the detector, V is the applied voltage, t_P_ the migration time of the protein (Aizpurua-Olaizola et al. 2018).

BGEs containing increasing concentrations of compound **1** and/or compound **2** were prepared at the pH for which μ_eff_ shifts were observed and used for K_D_ determination. For each carbohydrate and each studied concentration, the difference of the measured μ_eff_ of LecA with the μ_eff_ obtained without carbohydrate was determined. The obtained values were plotted vs. the carbohydrate concentration. Plots were fitted using the software Prism 8.0 (GraphPad Software Inc., La Jolla, CA, USA) applying nonlinear regression and assuming a 1:1 binding stoichiometry according to equation 2:

(2) \begin{equation*} \Delta{\mu}_{\mathrm{eff}}=\frac{{\mathrm{B}}_{\mathrm{max}}{\mathrm{C}}_{\mathrm{r}}}{{\mathrm{K}}_{\mathrm{d}}+{\mathrm{C}}_{\mathrm{r}}} \end{equation*}

where Δμ_eff_ is the effective mobility difference of LecA obtained at a specific carbohydrate concentration compared to the one obtained when no carbohydrate was added to the BGE, B_max_ is the maximum mobility shift, C_r_ is the carbohydrate concentration, and K_d_ is the dissociation constant (Aizpurua-Olaizola et al. 2018). K_d_ values and their standard deviations were calculated by the software using the least squares fitting method.

The μ_eff_ of the protein–ligand complexes differs from the μ_eff_ of the intact proteins or ligands. As carbohydrates are neutral at physiological pH a derivatization step is required to charge and give them better detection properties. The LecA peak was seen to shift upon binding of a monovalent charged ligand (**1**, 500 μM) and a stronger binding divalent ligand reversed the effect through competition.

### Bio-layer interferometry

BLI measurements were carried out using an Octet RED 384 instrument (ForteBio). Biosensors were transferred into fresh assay buffer for 180 s to collect a baseline read. Compound **3** and compound **4** were immobilized on streptavidin (SA)-functionalized biosensors to maximum levels by incubation for 900 s at 1 μM (Guo et al. 2018). For each set of biosensors, an 180 s baseline in buffer alone was acquired followed by an 1800 s association step and an 1800 s dissociation step. All experiments were carried out using PBS (10 mM phosphate, 150 mM NaCl, pH 7.4) as the assay buffer at 298 K. Data were processed and analyzed and K_D_ values were determined using the global fitting procedures as implemented in Octet® system Data Analysis Software 9.0.0.48 (Pall ForteBio LLC, Menlo Park, CA, USA). Kinetic responses were fitted to a 1-site binding model to obtain values for association (k_on_) and dissociation (k_off_) rate constants. The K_D_ derived from kinetic fitting was calculated as k_off_/k_on_.

### Thermal shift assay

TSA was performed using a standard real-time PCR instrument. To monitor protein unfolding, the fluorescent dye Sypro orange was used. Sypro orange is an environmentally sensitive dye (Thermo Fischer 5000x in DMSO). The unfolding process exposes the hydrophobic region of proteins and results in a large increase in fluorescence, which is used to monitor the protein-unfolding transition (Lo et al. 2004). The TSA was performed with a BioRad CFX96 real-time PCR machine, and analysis for binding induced shifts in thermal transition was performed with Bio-Rad CFX Manager 3.1 Software provided by the manufacturer (BioRad, Hercules, CA, USA).

Galactose based inhibitors specifically bind to the binding pocket of the lectin. Ligands could stabilize LecA (0.5 and 1 mg/mL) upon binding and therefore the protein T_m_ might increase. The shift in T_m_ with an addition of the ligand (ΔT_m_) serves as a qualitative measure of protein stability and allows to determine whether a protein–ligand binding event occurs. We here show the effect of different carbohydrates on the thermal denaturation of LecA. Sypro orange conc, used: 320× from commercial 5000×).

### Native ESI–MS

To investigate the interaction between LecA and compounds **1** and **2**, we incubated these compounds with LecA and transferred the produced complex into a 150 mM ammonium acetate solution (pH 7.5) by applying 10 μM of the protein ligand complex in a ratio of 1:1 on a pre-equilibrated 6-kDa Micro Bio-Spin column (Bio-Rad). The protein was eluted by centrifugation at 1,000 × g and 4°C. MS measurements were performed in positive ion mode using an Electrospray Ionization Time-of-Flight instrument (LC-T; Micromass, Manchester, UK) equipped with a Z-spray nanoelectrospray ionization source. Samples were sprayed from in-house prepared borosilicate glass capillaries (Kwik-Fil; World Precision Instruments, Sarasota, FL) prepared on a P-97 puller (Sutter Instruments, Novato, CA, USA) and coated with a thin layer of gold by using an Edwards Scan coat six Pirani 501 sputter coater (Edwards Laboratories, Milpitas, CA, USA). Instrument settings were adjusted for optimal transmission of high mass ions. A capillary voltage of 1300 V was used, in combination with a sampling cone voltage of 60 V. The source backing pressure was elevated to approximately 6.7 mbar in order to promote collisional cooling. All TOF data were mass calibrated by using an aqueous solution of cesium iodide (25 mg·mL^−1^) and interpreted manually using MassLynx software (Waters, version 4.1).

## Data Availability

The data underlying this article will be shared on reasonable request to the corresponding author.

## Abbreviations

μ_eff_, effective electrophoretic mobility; K_D_, dissociation constant; BGE, back ground electrolyte; JIL, Jacalin lectin; pI, isoelectric point; T_m_, melting temperature; ESI–MS, electro spray ionization mass spectrometery
